# Lifestyle Can Exert a Significant Impact on the Development of Metabolic Complications and Quality Life in Patients with Inflammatory Bowel Disease

**DOI:** 10.3390/nu15183983

**Published:** 2023-09-14

**Authors:** Sandra García-Mateo, Samuel J. Martínez-Domínguez, Carla Jerusalén Gargallo-Puyuelo, María Teresa Arroyo Villarino, Viviana Laredo De La Torre, Beatriz Gallego, Erika Alfambra, Fernando Gomollón

**Affiliations:** 1 IBD Unit, Department of Gastroenterology, “Lozano Blesa” Clinical Hospital, 50009 Zaragoza, Spain; samuelmartinez94@hotmail.com (S.J.M.-D.); carlajerusalen@hotmail.com (C.J.G.-P.); tarroyo.salud.aragon@gmail.com (M.T.A.V.); vlaredodelatorre@gmail.com (V.L.D.L.T.); fgomollon@gmail.com (F.G.); 2 Aragón Health Research Institute (IIS Aragón), 50009 Zaragoza, Spain; bgallego@iisaragon.es (B.G.); ealfambra.due@gmail.com (E.A.); 3 School of Medicine, University of Zaragoza, 50009 Zaragoza, Spain

**Keywords:** inflammatory bowel disease, mediterranean diet, physical activity, metabolic syndrome, type 2 diabetes mellitus, metabolic dysfunction-associated steatotic liver disease

## Abstract

Inflammatory bowel diseases (IBDs) are associated with an increased risk of metabolic comorbidities. There is a lack of data regarding the relationship between lifestyle and metabolic diseases in IBD patients. A cross-sectional study on consecutive IBD outpatients was conducted. Adherence to the Mediterranean diet (MD) was assessed using a 14-item questionnaire from the PREDIMED study, and physical activity was evaluated using the GODIN-Leisure score. Body composition was studied based on body mass index and waist–hip ratio (WHR), while quality of life was assessed using a nine-item short questionnaire. Among the 688 evaluated IBD patients, 66% were overweight or obese, 72.7% did not lead an active lifestyle and 70.1% did not adhere to the MD. Metabolic syndrome was associated with age (OR = 1.07, *p* = 0.019), overweight/obesity (OR = 12.987, *p* < 0.001) and the inflammatory behavior of Crohn’s disease (OR = 6.172, *p* = 0.001). Type 2 diabetes mellitus or prediabetes was associated with age (OR = 1.063 *p* = 0.016), overweight/obesity (OR = 3.861, *p* < 0.001) and the inflammatory behavior of Crohn’s disease (OR = 4.716, *p* = 0.001). Overweight /obesity (OR = 5.494, *p* < 0.001), a high WHR (OR = 2.564, *p* = 0.005) and a non-active lifestyle (OR = 2.202, *p* = 0.0003) were associated with metabolic dysfunction-associated steatotic liver disease. Lifestyle, body composition and not solely systemic inflammation might exert a significant influence on the emergence of metabolic comorbidities such as MASLD, type 2 diabetes mellitus and metabolic syndrome in patients with IBD.

## 1. Introduction

Inflammatory bowel diseases (IBDs) including Crohn’s disease (CD) and ulcerative colitis (UC) are characterized by relapsing–remitting inflammatory periods which lead to a state of chronic and systemic inflammation. In this context, despite the increasing availability of new treatments in recent years, a significant percentage fail to achieve deep clinical remission [1].

A chronic and systemic inflammation state is involved in the pathogenesis of most obesity-related comorbidities recently associated with IBD such as early atherosclerosis [2], metabolic syndrome, type 2 diabetes mellitus (DM2) [3] or metabolic dysfunction-associated steatotic liver disease (MASLD). While some authors have pointed out that the degree of activity, disease duration or corticosteroid use may favor the development of this metabolic condition, others focus their attention on well-stablished risk factors such as age or obesity, as is the case in the general population [4,5]. Even though obesity rates in patients with IBD seem to be similar to those observed in the general population, in recent decades, a parallel increas in both obesity and the incidence and prevalence of IBD as well as its metabolic-associated diseases have been noticed.

It is postulated that one of the links that could explain the pathophysiological association between IBD, obesity and its metabolic comorbidities in IBD patients could be the alterations in the intestinal microbiota. Dysbiosis has been demonstrated to induce and perpetuate intestinal inflammation with multiple harmful effects to host homeostasis, including the increase in intestinal permeability and the release of proinflammatory cytokines into systemic circulation [6]. Nevertheless, other often underestimated factors in the context of IBD management such as lifestyle can also play a crucial role [7]. In this line, some of the factors associated with the increased prevalence of IBD are common to the development of obesity and its metabolic complications, such as physical activity or dietary factors like high-fat diets or low-fiber diets [8].

Physical activity, nutritional habits and, above all, the combination of both as part of a healthy life represent key factors often overlooked in the management of chronic diseases and in the development of a healthy gut microbiome.

Despite the increased prevalence of metabolic complications arising from increased obesity among patients with IBD, studies on the impact of lifestyle on their diagnosis as well as risk factors are scarce.

The primary endpoint of this study was to evaluate the relationship of IBD course with patients’ lifestyles, and their relationship to metabolic complications such as overweight, obesity, MASLD, metabolic syndrome (MS) and DM2. The secondary endpoint of this study was to assess the quality of life based on lifestyle factors.

## 2. Materials and Methods

### 2.1. Study Population

This is a single-center, cross-sectional study. The study population comprised all IBD outpatients who agreed to participate and were followed at the IBD Unit of the University Hospital “Lozano Blesa”, in Spain, between October 2019 and April 2020.

The inclusion criteria were a confirmed diagnosis of IBD (UC or CD) with clinical follow-up for over a year and the signing of informed consent.

The following exclusion criteria were applied: individuals under 18 years of age, those without a confirmed diagnosis of UC or CD, patients diagnosed with IBD for less than 1 year and individuals with common causes of chronic liver disease other than MASLD, such as viral hepatitis or high-risk alcohol intake (>20 g per day for women and >30 g for men).

Out of 1068 patients who were initially considered, 310 declined to participate. Additionally, 8 patients were found to have non-determinate colitis, 6 patients had their IBD diagnosis excluded, 10 patients had a high alcohol consumption, 7 patients had a chronic hepatitis B virus infection and 25 had previously experienced a hepatitis B virus infection that did not progress to the chronic stage. Furthermore, 14 patients did not complete some of the questionnaires, resulting in a final sample of 688 patients for analysis, which was considered representative of the entire IBD cohort seen in our unit during a year (approximately 1200 patients).

### 2.2. Data Collection

After routine clinical evaluation at the IBD Unit, the patients who agreed to participate in the study were scheduled for a second assessment. The assessment consisted of two parts.

In the first one, all data related to their IBD were collected: IBD type (CD and UC), CD behavior (inflammatory (B1), stricturing (B2) and penetrating (B3)) [9], CD location (ileal (L1), colonic (L2), ileocolonic (L3), upper tract involvement (L4)), the existence of perianal disease (*p*) and the extension of UC (proctitis, left-side colitis or extensive colitis) were categorized according to the Montreal classification [10]. All data regarding the course of the disease were also collected, including the duration of the disease, considering the long-term evolution of the disease, starting from 10 years after the diagnosis. We also took into account the surgical requirements of the patients and the number of interventions they underwent, corticosteroid dependency as well as the number of systemic corticosteroid courses in the past year and in the last 5 years. Additionally, information on current treatments was also gathered. Clinical activity data were also collected through the Harvey-Bradshaw index (in CD, >4 points was considered active) or partial Mayo index (in UC, >2 points) [11].

In the second part, an assessment of comorbidities and lifestyle was performed. The presence of DM2 was considered if fasting plasma glucose is equal or greater than 126 mg/dL or if the glycated hemoglobin (HbA1) level is equal or greater than 6.5%, whereas prediabetes was considered with fasting plasma glucose values between 100 and 125 or HbA1 between 5.7 and 6.4% [12]. After excluding other causes of chronic liver disease, and in conformity with recent published criteria [13], MASLD diagnosis was given if steatotic liver disease was associated with either a BMI > 25 kg/m^2^ or waist circumference ≥ 102/88 cm, blood pressure ≥ 130/85 mmHg or specific antihypertensive drug treatment, plasma triglycerides ≥ 150 mg/dL and plasma HDL-cholesterol ≤ 40 mg/dL for males and ≤50 mg/dL for females [14]. Steatotic liver disease was diagnosed via both ultrasonography and controlled attenuation parameter (CAP^TM^) > 248 dB/min.

For metabolic syndrome, diagnosis was based on the National Cholesterol Education Program’s Adult Treatment Panel III (NCEP ATP3 criteria) [15], where ≥3 factors are required.

A comprehensive anthropometric assessment of the patients was also conducted. The body mass index (BMI) was considered underweight if <18 kg/m^2^, normal if 18.5–25 kg/m^2^, overweight > 25–29.9 kg/m^2^ and obese if BMI ≥ 30 kg/m^2^. The waist circumference (WC) was considered high if ≥102 cm for men and ≥88 cm for women. The waist–hip ratio (WHR) was considered increased if >0.8 (women) and >0.9 (men).

Finally, through the completion of two validated questionnaires, the lifestyle of the patients was assessed. Firstly, adherence to MD was evaluated using the 14-item “Prevención con dieta mediterránea” (PREDIMED) questionnaire [16], where scores ≥ 9 indicated proper adherence. Secondly, a GODIN-Leisure physical activity score [17] of ≥ 24 correlated with an active lifestyle. We considered patients with good lifestyle habits as those with good adherence to MD and an active lifestyle. Moreover, a 9-item short questionnaire (IBDQ-9) to assess patients’ quality of life was also administered.

### 2.3. Statistical Analysis

Firstly, an initial exploratory analysis of all variables was performed. Qualitative variables are expressed as frequencies and percentages, whereas quantitative ones are expressed as means ± standard deviation (SDs) or medians + interquartile ranges (IQRs). The normal distribution of the variables was assessed using the Kolmogorov–Smirnov test.

Qualitative variables were compared using the chi-square test. Medians between two independent groups were compared with the Mann–Whitney test and Kruskal–Wallis test (when the qualitative and nonparametric variables have more than two categories). Patients who did not correctly complete all the questionnaires were excluded in order to avoid errors stemming from incomplete data.

A binomial generalized linear model with logistic regression analysis was applied to study the impact of disease course and lifestyle on the existence of comorbidities. All the variables that reached statistical significance in the univariate analysis as well as those which were considered clinically relevant were included in the multivariate analysis. Logistic regression analysis generated a percentage per variable. The odds ratio (OR) can be interpreted as an exponential function of each coefficient. The statistical analysis was performed using SPSS version 26. For all tests, a two-sided *p*-value less than 0.05 was considered statistically significant.

## 3. Results

### 3.1. Study Population

A total of 688 patients were included. The main clinical characteristics of IBD patients, along with their anthropometric measurements and comorbidities, are presented in Table 1. Fifty-three percent of the patients had UC. The mean age was 49 (IQR 39–59) years old. Nineteen percent of the patients required abdominal surgery during the course of the disease, and the most commonly prescribed therapies at the time of inclusion in the study were 5-aminosalicylates (33.9%) followed by anti-TNF medications (22.5%). Moreover, 14.4% of the patients had been exposed to systemic steroids in the last year, 35.9% in the last 5 years, and 29.8% had a history of steroid dependence in the course of the disease.

A significant portion of the patients did not lead an active lifestyle (72.7%) and were also not adherent to the MD (70.1%), resulting in only 63 patients (9.2%) following a healthy lifestyle.

### 3.2. Overweigh and Obesity in IBD Patients: Associated Factors

Most of the patients (51.5%) were found to have either overweight (34.6%) or obesity (16.9%).

In the univariate analysis, the presence of overweight and obesity showed a significant association with male sex, older age, higher age at diagnosis, as well as having a higher WHR and being an ex-smoker. None of the disease-related characteristics, except for the absence of clinical activity, showed a significant association with the development of overweight or obesity (Table 2).

Considering the significant variables in the univariate analysis, only age (OR = 1.026 95% CI = 1.006–1.047, *p* = 0.011), high WHR (OR = 2.808, 95% CI = 1.464–5.405, *p* = 0.002) and non-adherence to MD (OR = 0.599, 95% CI = 0.373–0.962, *p* = 0.034) were associated with overweight after logistic regression was performed.

On the other hand, male sex (OR = 2.724 95% CI = 1.113–6.666, *p* = 0.028), high WHR (OR = 5.780, 95% CI = 2.358–14.285, *p* < 0.001) and the absence of clinical disease activity (OR = 3.030 95% CI = 1.226–7.407, *p* = 0.016) were associated with obesity in multivariate analysis.

### 3.3. Metabolic Comorbidities of Overweight and Obesity in IBD Patients: Associated Factors

Regarding metabolic comorbidities, 24.4% of patients me the criteria for MS, 35.2% for DM2 or prediabetes, and 39% for MASLD.

In the univariate analysis, all these conditions were found to be associated with male gender, age, former smoking, overweight, obesity, high WHR and age at diagnosis. Moreover, inflammatory behavior of CD was associated with MS, DM2 and prediabetes, but not with MASLD. Additionally, the need for any steroid therapy in the last 5 years was only associated with DM2 and prediabetes but not with MS or MASLD (Table 3).

Taking all these variables into account in the multivariate analysis, MS was statistically associated with age (OR = 1.07, 95% CI = 1.011–1.133, *p* = 0.019), overweight or obesity (OR = 12.987, 95% CI = 5.586–30.303, *p* < 0.001) and the inflammatory behavior of CD (OR = 6.172, 95% CI = 2.105–18.181, *p* = 0.001). Similarly, DM2 or prediabetes were associated with age (OR = 1.063 95% CI = 1.011–1.118, *p* = 0.016), overweight or obesity (OR = 3.861, 95% CI = 2.061–7.246, *p* < 0.001) and the inflammatory behavior of CD (OR = 4.716, 95% CI = 1.865–11.904, *p* = 0.001). The presence of overweight or obesity (OR = 5.494, 95% CI = 3.759–8.064, *p* < 0.001), high WHI (OR = 2.564, 95% CI = 1.324–4.950, *p* = 0.005) and a non-active lifestyle (OR = 2.202, 95% CI = 1.319–3.676, *p* = 0.0003) were associated with a diagnosis of MASLD.

### 3.4. IBD Characteristics, Metabolic Comorbidities, and Their Relationship with Lifestyle

As previously mentioned, over half of the patients were overweight or obese; however, the proportion of patients who experienced metabolic complications was lower. To gain a better understanding of the causal relationship, their association with IBD characteristics and metabolic complications with lifestyle was analyzed.

In univariate analysis, it was observed that women, older patients, and those with lower BMI and WHR were more likely to adhere to the MD. The only variables associated with not having an active lifestyle were lower levels of HDL cholesterol and being overweight or obese. When considering a healthy lifestyle (both physical activity and adherence to MD), the only variable associated was obesity or overweight (Appendix A).

**Figure 1:**
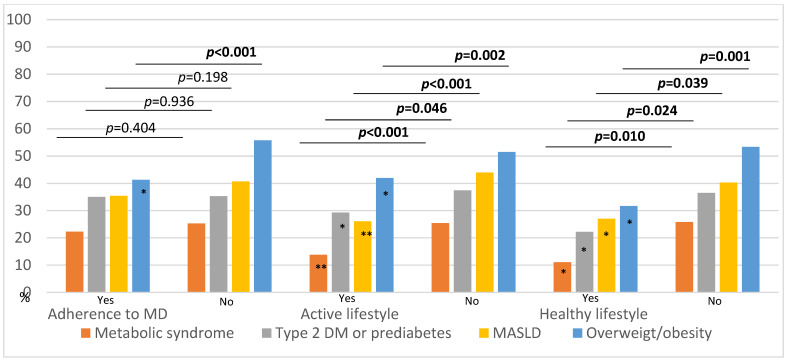
Relationship between lifestyle and overweight, obesity and its metabolic comorbidities in patients with IBD. Healthy lifestyle: considering both adherence to Mediterranean diet and active lifestyle. MS: metabolic syndrome. DM2: type 2 diabetes mellitus. MASLD: metabolic dysfunction-associated steatotic liver disease. * Significative variables ** Significative variables (<0.001).

Although a protective trend in adherence to the MD ant its association with a lower likelihood of being diagnosed with MASLD, MS, DM2 and prediabetes is evident, the statistical significance of MD adherence was only found with the overweight or obesity condition. However, both an active lifestyle, as assessed using the DOGIN questionnaire, and a healthy lifestyle (defined as both physical activity and adherence to the MD) were significantly associated with a lower likelihood of being diagnosed with overweight or obesity, MASLD, MS, DM2 and prediabetes (Figure 1).

Among all the variables analyzed, only the lack of an active lifestyle and non-adherence to the MD were found to be significantly associated with the development of MASLD and overweight or obesity, respectively, in the multivariate analysis, as mentioned before.

### 3.5. Impact of Lifestyle on the Quality of Life of Patients with IBD

The relationship between IBDQ-9 score and lifestyle was assessed. Patients with correct adherence to the MD showed a higher mean score of 51 (IQR 47–56) compared to those who were non-adherent, who scored a mean of 50 (IQR 46–54), but statistical significance was not reached.

**Figure 2:**
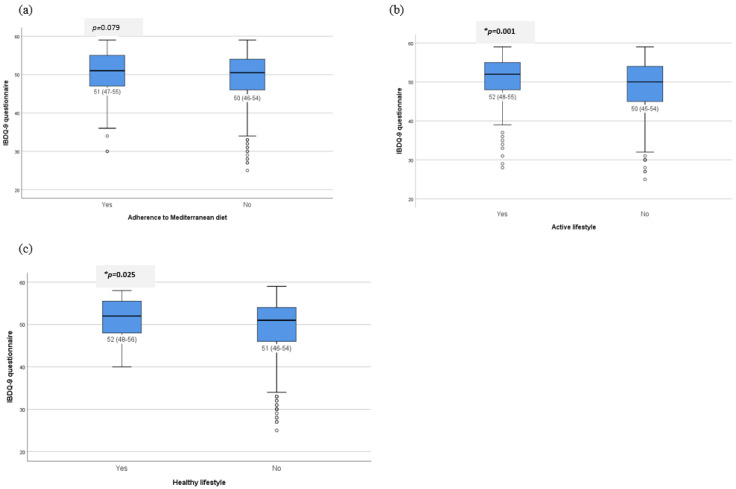
Relationship between lifestyle: (**a**) adherence to Mediterranean diet, (**b**) active lifestyle and (**c**) healthy lifestyle (adherence to Mediterranean diet and active lifestyle) with nine-item short IBD questionnaire (IBDQ-9). Results of IBDQ-9 questionnaire are expressed as mean (interquartile range). * Univariate analysis.

However, both patients with an active lifestyle, as determined using the GODIN questionnaire (with a mean score of 52 (IQR (48–55)), and those with a healthy lifestyle (with a mean score of 52 IQR (48–56)) had significantly higher IBDQ-9 scores when compared to those without an active lifestyle and non-healthy lifestyle, as is shown in Figure 2.

## 4. Discussion

The incidence of IBD has risen globally in conjunction with the obesity epidemic as well as its associated metabolic diseases like MS, DM2 and MASLD [8,18,19].

In our cohort of 688 IBD outpatients, 34.6% were overweight and 16.9% were obese. The data align with findings from other cross-sectional studies, such as those by Lomer et al. [20] and pringle et al. [21] with overweight rates of 29–30% and obesity rates of 18–16%, respectively.

While previous studies have shown an association between obesity and UC rather than CD [22], we did not observe these results in our cohort, and although the proportion of UC patients with overweight (55.9%) and obesity (51.7%) was higher than that of CD patients, it did not reach statistical significance (*p* = 0.451 and *p* = 0.857, respectively). Similarly, we did not find any differences between the presence of obesity or overweight and the location of a pattern of CD as described in other cohorts [21]. We observed a significant association between smoking cessation and overweight and obesity, as has been reported previously. In fact, smoking habits have been considered as a strategy to control or lose weight among healthy younger adults and those smokers who experienced weight gain after previous attempts to quit [23]. While some individuals have used smoking as a perceived means to control weight, it is essential to stress its detrimental health effects. For IBD patients, particularly those with CD, avoiding smoking is paramount due to its association with a worse prognosis of the disease [24]. Balancing the potential risk of weight gain with the known negative effects of smoking in IBD, efforts to support patients in achieving a healthy lifestyle and weight management while quitting smoking are of utmost importance.

In our cohort, patients with stable disease had a higher prevalence of obesity compared to those with disease activity. Although the use of corticosteroids in the general population has been associated with weight gain and increased appetite [25], patients with IBD experiencing an active flare, even when taking corticosteroids, are at risk of weight loss and malnutrition, as has been previously described [26].

According to our findings, advancing age and male sex have been identified as independent risk factors for overweight and obesity, respectively. This association between aging, male sex and obesity may be explained by the decrease in testosterone levels over time in men, as well as the decline in skeletal muscle mass accompanied by an accumulation of visceral adipose tissue as individuals age [27]. Furthermore, aging (especially over 50 years) is a significant contributor to metabolic deterioration, including the development of insulin resistance and MS [28]. In fact, aging, which is characterized by chronic, low-grade systemic inflammation due to immune system dysregulation, also contributes to the onset and progression of metabolic diseases. Therefore, in our IBD patients, not only overweight and obesity but also the development of MS (OR = 1.07, 95% CI = 1.011–1.133, *p* = 0.019), DM2 and prediabetes (OR = 1.063, 95% CI = 1.011–1.118, *p* = 0.016) has been associated with aging, as observed in the general population.

High WHR was also related to the development of overweight (OR = 2.808, 95% CI = 1.464–5.405, *p* = 0.002), obesity (OR = 5.780, 95% CI = 2.358–14.285, *p* < 0.001) and MASLD (OR = 2.564, 95% CI = 1.324–4.950, *p* = 0.005) in our IBD patients, as has been described in other cross-sectional studies for both IBD (5) and non-IBD patients [14]. The distribution of body fat is more critical than BMI when considering metabolic comorbidities. It has been demonstrated that the percentage of visceral fat in humans can have a stronger association with metabolic alterations and increased insulin resistance [28], which is one of the pathophysiological foundations for the development of MASLD [13,14,29].

In line with this, the inflammatory behavior of CD has been associated with MS and DM2 based on our findings. Central obesity, which reflects visceral fat accumulation, is associated with proinflammatory states and MS. In fact, it is well known that chronic inflammation states may induce dyslipidemia through the action of proinflammatory cytokines such as tumor necrosis factor- α or interleukin-6 perpetuating this visceral fat accumulation. Although overweight and obesity has been rising in the IBD population, CD has been related with so-called “creeping fat”, which is the effect of local hyperplasia in the excess of visceral adipose tissue surrounding the abdominal organs and the inflamed intestine. This creeping fat is associated not only with transmural inflammation but also with fibrosis and stricture patterns, which may contribute to perpetuating proinflammatory states and MS [30].

As in other cross-sectional studies performed in IBD patients [22], overweight and obesity have been related to the onset of metabolic complications such as MAFLD, DM2 or prediabetes as happens in the general population. However, according to Rodriguez-Duque J et al. [5], when comparing IBD and non-IBD patients matched by sex and age, MALFD was significantly more prevalent in the IBD population.

Although the chronic inflammatory status and dysbiosis of IBD patients may explain the development of chronic metabolic diseases, lifestyle also play a crucial role according to our results.

In that line, based on our results, MD plays a protective role against overweight (OR = 0.599, 95% CI = 0.373–0.962, *p* = 0.034). In fact, its protective role in the primary prevention of cardiovascular events has been shown in healthy Spanish people [16]. However, good nutritional habits such as adhering to the MD are scarce among the population with IBD. On many occasions, changes in nutritional habits occur after the diagnosis of the disease. There are several reasons why patients may change their dietary patterns and may not adhere to the MD, and this could be related to the fact that they associate certain foods, such fiber, with a higher likelihood of flare-ups, a fear of worsening symptoms, or the avoidance of some food groups during a flare-up, which isan independent risk factor for the development of malnutrition [26]. However, Chicco F et al. have demonstrated an improvement of malnutrition status as well as a reduction in disease activity index and liver steatosis parameters in both CD and UC patients after a short-term MD dietary intervention [7]. Although it did not reach statistical significance, the diagnosis of MASLD, DM2, prediabetes and MS occurred more often among IBD patients without a correct adherence to MD, as had been reported previously [31].

Despite the numerous benefits of physical activity associated with reduced risk of all-cause mortality with or without previous CV events [32], many patients with IBD are severely limited in this regard, not only due to periods of disease activity, the need for surgery or therapy escalation but also because of the chronic fatigue states that many patients experience, even when they are in clinical remission [33]. Moreover, other non-physical factors may also contribute, such as the higher prevalence of depression and mood disturbances among this population. In fact, approximately 1/3 of patients experience a reduction in their physical activity level after IBD diagnosis [34], with an increased likelihood of obesity and its associated metabolic complications. This could potentially explain the low rate of physical activity in our cohort. In this way, the increase in sedentary behavior, which is becoming a growing problem in the general population, could be even higher in IBD patients. In our cohort, physical activity was related with a significatively lower prevalence of overweight, obesity, MS, DM2 and MASLD with statistical significance regarding the development of MASLD in the multivariate analysis (OR = 2.202, 95% CI = 1.319–3.676, *p* = 0.0003). These findings could partially explain the increased prevalence of MASLD among the population with IBD when compared to non-IBD controls [5].

When considering quality of life based on the IBDQ-9 questionnaire, a significant association with a healthy lifestyle and an active lifestyle was observed. As metabolic comorbidities are less frequently diagnosed in patients with a healthy lifestyle, it is logical to consider an association between a better quality of life in that context, as has been reported previously [7].

From the perspective of study limitations, this study is constrained by its single-center and cross-sectional design, which limits the ability to establish causal relationships and makes it challenging to assess the long-term effects of lifestyle factors on metabolic comorbidities in patients with IBD. Moreover, the assessment of adherence to the MD, as well as physical activity, was conducted through questionnaires administered to the patient on the day of the clinical interview. Through relying on patient-reported information for the results rather than being objectively observed by an external observer, the potential for information bias could have arisen. Finally, the presence of a control group would have enabled us to reach more robust conclusions. However, it is practically unfeasible to locate a comparable control group without IBD, given the constraints related to diet and physical activity that IBD patients face due to flare-ups and chronic fatigue, which may affect patients even in clinical remission.

It is important to highlight the strengths of this study. It consists of a cohort of well-characterized IBD outpatients. Moreover, to the best of our knowledge, this is the largest cross-sectional cohort study that examines the relationship between lifestyle factors, including adherence to the MD and physical exercise, and highly prevalent yet underdiagnosed comorbidities such as MASLD, DM and MS in patients with IBD, which enhances its representativeness and generalizability to the broader population of individuals with IBD. Additionally, the diagnosis of MASLD was conducted using CAP rather than analytical scores, which enhances the reproducibility and robustness of the results. This study aims to provide a better understanding of the development of these metabolic comorbidities in patients with IBD, incorporating both clinical and lifestyle-related aspects.

## 5. Conclusions

To conclude, despite the chronic inflammatory activity inherent in patients with IBD, lifestyle factors such as diet, physical activity and smoking cessation play a crucial role in their overall health profile.

## Figures and Tables

**Table 1:** General characteristics, comorbidities, anthropometrics and clinical variables of IBD patients.

General Characteristic, Comorbidities, and Anthropometric Variables	Total (n = 688)	Clinical Variables Related to the Disease	Total (n = 688)
Sex: male n (%)	341 (49.6)	Age of diagnosis, mean (IQR)	35 (26–45)
Age, median (IQR)	49 (39–59)	Years of evolution of the disease, mean (IQR)	11 (5–20)
Current smokers: yes, n (%):	137 (80.1)	More than 10 years of evolution, n (%)	394 (57.4)
Former smokers, yes, n (%):	313 (45.5)	IBD type and Location, n (%) UC Proctitis (UC) Left side colitis (UC) Extensive colitis (UC) CD Ileal (CD, L1) Ileocolonic (CD, L3) Colonic (CD, L2)	369 (53.6) 91 (24.6) 137 (37.1) 141 (38.2) 319 (46.4) 124 (38.8) 150 (47) 45 (14.1)
Chronic renal disease: yes, n (%):	65 (9.4)	Behaviour (EC), n (%) Inflammatory (B1) Stricturing (B2) Penetrating (B3)	194 (60.8) 85 (26.6) 43 (13.5)
Cerebrovascular disease: yes, n (%) Cardiovascular disease: yes, n (%)	10 (1.5) 25 (3.6)	Perianal disease, yes n (%)	86 (12.5)
SBP, median (IQR) DBP, median (IQR) Hypertension: yes, n (%):	132 (121–148) 80 (72–86) 106 (15.4)	Extraintestinal disease, yes n (%)	136 (19.8)
Body mass Index, n (%): Low weight Normal weight Overweight Obesity	15 (2.2) 319 (46.4) 238 (34.6) 116 (16.9)	Need for surgery, yes n (%)	133 (19.3)
Waits to hip ratio: high n (%):	344 (50)	Threartment, n (%) None 5 ASA Anti TNF Anti TNF and IMS IMS Vedolizumab Ustekinumab Tofacitinib Esteroids	95 (13.8) 233 (33.9) 155 (22.5) 34 (4.9) 75 (10.9) 32 (4.7) 49 (7.1) 5 (0.7) 10 (1.5)
Metabolic syndrome, yes n (%)	168 (24.4)	Steroid dependence, yes, n (%)	208 (29.8)
MASLD, yes, (%)	269 (39.1)	Systemic steroids in the last year, yes (n%)	99 (14.4)
DM2 or pre-DM: yes, n (%): Insulin, m (IQR)	242 (35.2) 6.95 (4.1–12.3)	Systemic steroids in the last 5 years, yes (n%)	247 (35.9)
High total cholesterol, n (%):	188 (165–210)	Topical steroids in the last year, yes (n%)	8 (1.2)
Low HDL, n (%):	114 (16.6)	Topical steroids in the last 5 years, yes (n%)	25 (3.6)
Triglycerides: high, n (%):	132 (19.2)	Disease activity, yes, n (%)	41 (6)

CD: Crohn’s disease. UC: ulcerative colitis. m: mean. IQR: interquartile range. 5 ASA: 5-aminosalicylates. IMMs: immunomodulators. MD: Mediterranean diet. SBP: systolic blood pressure. DBP: diastolic blood pressure. DM2: diabetes mellitus. HDL: high-density lipoprotein. MASLD: metabolic dysfunction-associated steatotic liver disease.

**Table 2:** Overweight and obesity in IBD patients and associated factors.

	No Overweight/Obesity (n = 234)	Overweight Yes (n = 238)	*p*-Value *	Obesity Yes (n = 116)	*p*-Value *
Sex; females n (%)	200 (59.9)	94 (39.5)	<0.001	53 (45.7)	0.008
Age; m (IQR)	46 (36–57)	53 (41–61.5)	<0.001	52.5 (44–60)	0.001
Smoker; n (%)	77 (23.1)	39 (16.4)	0.051	21 (18.1)	0.266
Former smoker; n (%)	127 (38)	123 (51.7)	0.001	63 (54.3)	0.002
High WHR; n (%)	120 (36.1)	151 (63.4)	0.001	73 (62.9)	0.001
Age at diagnosis, m (IQR)	31.5 (24–42)	37 (29–47)	<0.001	37 (29–48)	0.001
More than 10 years of evolution; Yes, n (%)	189 (56.6)	141 (59.4)	0.488	64 (55.2)	0.791
IBD type; UC n (%)	176 (52.7)	133 (55.9)	0.451	60 (51.7)	0.857
Location; n (%) Proctitis (UC) Left side colitis (UC) Pancolitis (UC) Ileal (L1, CD) Ileocecal (L3, CD) Colonic (L2, CD)	42 (12.6) 65 (19.5) 69 20.7) 64 (19.2) 71 (21.3) 23 (6.9)	35 (14.7) 46 (19.3) 52 (21.8) 43 (18.1) 51 (21.4) 11 (4.6)	0.869	14 (12.1) 26 (22.4) 20 (17.2) 17 (14.7) 28 (24.1) 11 (9.5)	0.706
Behavior (CD); n (%) Inflammatory (B1) Stricturing (B2) Penetrating (B3)	103 (65.2) 35 (22.2) 20 (12.7)	57 (54.3) 35 (33.3) 13 (12.4)	0.122	34 (60.7) 13 (23.2) 9 (16.1)	0.777
Perianal disease; yes n (%)	42 (26.6)	25 (23.8)	0.613	19 (33.9)	0.295
Clinical remission; yes n (%)	13 (3.9)	16 (6.7)	0.128	12 (10.3)	0.009
Extraintestinal disease; yes n (%)	70 (21)	43 (18.1)	0.392	23 (19.8)	0.796
History of surgery; yes n (%)	62 (18.6)	43 (18.1)	0.880	28 (24.1)	0.196
More than two surgeries, n (%)	32 (9.6)	15 (6.3)	0.159	9 (7.8)	0.557
Need of any biologic therapy; yes n (%)	78 (27.4)	56 (26.9)	0.913	29 (29)	0.754
Steroid dependence; yes n (%)	105 (31.8)	65 (27.7)	0.288	35 (30.4)	0.783
Need of any steroid therapy in the last 5 years, n (%)	130 (39.4)	81 (34.5)	0.233	49 (42.6)	0.545
IBDQ-9	51 (45–54.3)	51 (47–55)	0.412	50.5 (46–54)	0.860
Adherence to MD, n (%)	121 (36.2)	59 (24.8)	0.004	26 (22.4)	0.006
Active lifestyle, n (%)	109 (32.6)	59 (24.8)	0.042	20 (17.2)	0.002
Healthy lifestyle, n (%)	43 (12.9)	15 (6.3)	0.010	5 (4.3)	0.010

IBD: inflammatory bowel disease. WHR: waist–hip ratio. m: mean. IQR: interquartile range. UC: ulcerative colitis. IBDQ-: inflammatory bowel disease questionnaire short form. MD: Mediterranean diet. * Univariate analysis.

**Table 3:** Metabolic comorbidities in IBD patients and associated factors.

	Metabolic Syndrome		*p*-Value	Type 2 DM or Prediabetes		*p*-Value	MASLD		*p*-Value
	No (n = 520)	Yes (n = 168)		No (n = 446)	Yes (n = 242)		No (n = 419)	Yes (n = 269)	
Sex; females n (%)	274 (52.7)	73 (43.5)	0.037	258 (57.8)	89 (36.8)	<0.001	252 (60.1)	95 (35.3)	<0.001
Age; m (IQR)	46 (37–57)	58 (50–65.8)	<0.001	46 (36.5–56)	55 (46–64)	<0.001	46 (36–57)	54 (44.5–62.5)	<0.001
Smoker; yes n (%)	105 (20.2)	32 (19)	0.747	95 (21.3)	42 (17.4)	0.216	88 (21)	49 (18.2)	0.372
Former smoker; yes n (%)	221 (42.5)	92 (54.8)	0.006	185 (41.5)	128 (52.9)	0.004	178 (57.5)	135 (50.2)	0.048
Overweight or obesity (BMI); n (%) Yes	207 (39.8)	147 (87.5)	<0.001	186 (41.7)	168 (69.4)	<0.001	143 (34.1)	211 (78.4)	<0.001
Waist hip ratio; high n (%)	230 (44.4)	114 (67.9)	<0.001	183 (41.1)	161 (66.8)	<0.001	155 (37.2)	189 (70.3)	<0.001
Age at diagnosis, mean (IQR)	33 (25–42)	42 (32.3–50.8)	<0.001	32 (25–42)	38 (30–49)	<0.001	32 (25–43)	37 (29.5–47)	<0.001
More than 10 years of evolution; Yes, n (%)	285 (54.9)	109 (64.9)	0.023	237 (53.3)	157 (64.9)	0.003	223 (53.3)	171 (63.6)	0.008
IBD type; UC n (%)	285 (54.8)	84 (50)	0.277	232 (52)	137 (56.6)	0.249	231 (55.1)	138 (51.3)	0.326
Location; n (%) Proctitis (UC) Left side (UC) Pancolitis (UC) Ileal (CD) Ileocecal (CD) Colonic (CD)	70 (13.5) 101 (19.4) 115 (22.1) 97 (18.7) 108 (20.8) 29 (5.6)	21(12.5) 36 (21.4) 26 (15.5) 27 (16.1) 42 (25) 16 (9.5)	0.179	58 (13) 81 (18.2) 93 (20.9) 87 (19.5) 101 (22.6) 26 (5.8)	33 (13.6) 56 (23.1) 48 (19.8) 37 (15.3) 49 (20.2) 19 (7.9)	0.413	61 (14.6) 83 (19.8) 88 (21) 74 (17.7) 88 (21) 25 (6)	30 (11.2) 54 (20.1) 53 (19.7) 50 (18.6) 62 (23) 20 (7.4)	0.775
Behavior (CD); n (%) Inflammatory (B1) Stricturing (B2) Penetrating (B3)	142 (60.4) 68 (28.9) 25 (10.6)	52 (61.9) 15 (17.9) 17 (20.2)	0.026	133 (62.1) 62 (29) 19 (8.9)	61 (58.1) 21 (20) 23 (21.9)	0.003	118 (62.8) 47 (25) 23 (12.2)	76 (58) 36 (27.5) 19 (14.5)	0.680
Perianal disease; yes n (%)	60 (25.5)	26 (31)	0.337	51 (23.8)	35 (33.3)	0.072	52 (27.7)	34 (26)	0.736
Clinical remission; yes n (%)	492 (94.6)	155 (92.3)	0.263	420 (94.2)	227 (93.8)	0.845	397 (94.7)	250 (92.9)	0.327
Extraintestinal disease; yes n (%)	103 (19.8)	33 (19.6)	0.963	89 (20)	47 (19.4)	0.867	82 (19.6)	54 (20.1)	0.871
History of surgery; yes n (%)	93 (17.9)	40 (23.8)	0.091	83 (18.6)	50 (20.7)	0.515	72 (17.2)	61 (22.7)	0.075
Need of any biologic therapy; yes n (%)	129 (28.2)	34 (25)	0.459	106 (27.7)	57 (27)	0.848	96 (26.3)	67 (29.4)	0.413
Anti-TNF therapy; n (%) Yes	129 (28.9)	34 (25)	0.380	106 (28.3)	57 (27.3)	0.783	96 (26.7)	67 (29.9)	0.407
Steroid dependence; yes n (%)	162 (31.5)	43 (26.1)	0.189	143 (32.4)	62 (25.9)	0.079	124 (29.7)	81 (30.8)	0.769
Need of any steroid therapy in the last 5 years. Yes n (%)	199 (38.6)	61 (37)	0.701	186 (42.2)	74 (31)	0.004	159 (38.2)	101 (38.3)	0.992
IBQ-9; m(IQR)	51 (46–54)	51 (45–54)	0.650	51 (46–54)	51 (46–55)	0903	51 (46–54)	51 (46–54)	0.593

DM: diabetes mellitus. m: mean. IQR: interquartile range. IBDQ-9: inflammatory bowel disease questionnaire short form. MASLD: metabolic dysfunction-associated steatotic liver disease.

## Data Availability

All data are available in the main text and Appendix A.

## Appendix A

**Table A1:** Relationship between lifestyle to disease and anthropometric characteristics in IBD patients.

	Adherence to MD		*p*-Value	Active Lifestyle		*p*-Value	Healthy Lifestyle *		*p*-Value
	Yes (n = 206)	No (n = 282)		Yes (n = 188)	No (n = 500)		Yes (n = 63)	No (n = 625)	
Sex; females n (%)	124 (60.2)	223 (46.3)	0.001	93 (49.5)	254 (50.8)	0.756	36 (57.1)	311 (49.8)	0.264
Age; m (IQR)	53 (42–62)	47 (38–58)	0.001	47 (37–58.8)	50 (40–59)	0.161	53 (42–61)	48 (39–59)	0.133
Smoker; yes n (%)	39 (18.9)	98 (20.3)	0.674	31 (16.5)	106 (21.2)	0.168	11 (17.5)	126 (20.2)	0.609
Former smoker; yes n (%)	101 (49)	212 (44)	0.224	82 (43.6)	231 (46.2)	0.544	32 (50.8)	281 (45)	0.375
WHR; high n (%)	82 (39.8)	262 (54.6)	<0.001	88 (47.3)	256 (51.2)	0.365	25 (39.7)	319 (51.2)	0.081
Hypertension; yes n (%)	40 (19.4)	66 (13.7)	0.057	23 (12.2)	83 (16.6)	0.157	11 (17.5)	95 (15.2)	0.636
Renal disease; yes n (%)	14 (7)	51 (10.8)	0.126	18 (9.8)	47 (9.6)	0.912	7 (11.3)	58 (9.5)	0.642
Cerebrovascular disease, yes n (%)	2 (1)	8 (1.7)	0.731	2 (1.1)	8 (1.6)	0.601	0 (0)	10 (1.6)	0.312
CV disease; yes n (%)	10 (4.9)	15 (3.1)	0.263	5 (2.7)	20 (4)	0.402	2 (3.2)	23 (3.7)	0.838
HDL cholesterol; low n (%)	28 (13.6)	86 (17.8)	0.170	22 (11.7)	92 (18.4)	0.035	6 (9.5)	108 (17.3)	0.115
Age at diagnosis, mean (IQR)	36 (28–48)	34 (25–44)	0.003	34 (25–45)	36 (27–45)	0.260	36 (29–49)	35 (26–45)	0.088
More than 10 years of evolution; Yes, n (%)	122 (59.2)	272 (56.5)	0.516	107 (56.9)	287 (57.5)	0.887	35 (55.6)	359 (57.5)	0.762
IBD type; UC n (%)	118 (57.3)	251 (52.1)	0.210	109 (58)	260 (52)	0.161	41 (65.1)	328 (52.5)	0.056
Location; n (%) Proctitis (UC) Left side (UC) Pancolitis (UC) Ileal (L1, CD) Ileocecal (L3, CD) Colonic (L2, CD)	35 (17) 37 (18) 47 (22.8) 41 (19.9) 32 (15.5) 14 (6.8)	56 (11.6) 100 (20.7) 94 (19.5) 83 (17.2) 118 (24.5) 31 (6.4)	0.065	31 (16.5) 37 (19.7) 41 (21.8) 30 (16) 40 (21.3) 9 (4.8)	60 (12) 100 (20) 100 (20) 94 (18.8) 110 (22) 36 (7.2)	0.525	12 (19) 15 (32.8) 14 (22.2) 8 (12.7) 12 (19) 2 (3.2)	79 (12.6) 122 (19.5) 127 (20.3) 116 (18.6) 138 (22.1) 43 (6.9)	0.423
Behavior (CD); n (%) Inflammatory Stricturing Penetrating	62 (70.5) 19 (21.6) 7 (8)	132 (57.1) 64 (27.7) 35 (15.2)	0.071	53 (67.1) 17 (21.5) 9 (11.4)	141 (58.8) 66 (27.5) 33 (13.8)	0.417	18 (81.8) 4 (18.2) 0 (0)	176 (59.3) 79 (26.6) 42 (14.1)	0.068
Perianal disease; yes n (%)	18 (20.5)	68 (29.4)	0.106	20 (25.3)	66 (27.5)	0.704	4 (18.2)	82 (27.6)	0.336
Clinical remission; yes n (%)	4 (4.5)	14 (6.1)	0.600	2 (2.5)	16 (6.7)	0.167	0 (0)	18 (6.1)	0.235
Extraintestinal disease; yes n (%)	37 (18)	99 (20.5)	0.437	34 (18.1)	102 (20.4)	0.497	9 (14.3)	127 (20.3)	0.252
History of surgery; yes n (%)	26 (12.6)	107 (22.2)	0.004	29 (15.4)	104 (20.8)	0.112	7 (11.1)	126 (20.2)	0.083
Need of any biologic therapy; yes n (%)	38 (22.4)	125 (296)	0.076	39 (23.2)	124 (29.2)	0.143	10 (18.9)	153 (28.3)	0.141
Anti-TNF therapy; n (%) Yes	38 (22.8)	125 (30)	0.076	39 (23.8)	124 (29.6)	0.160	10 (19.2)	153 (28.8)	0.142
Steroid dependence; yes n (%)	51 (25)	154 (32.4)	0.056	47 (25.1)	158 (32)	0.079	13 (21)	192 (31.1)	0.099
Need of any steroid therapy in the last 5 years. Yes n (%)	70 (34.1)	190 (40)	0.149	70 (37.8)	190 (38.4)	0.896	21 (33.9)	239 (38.7)	0.458

* Healthy lifestyle: adherence to Mediterranean diet and active lifestyle. IQR: interquartile range. WHR: waist–hip ratio. CV: cardiovascular. HDL: high-density lipoprotein. UC: ulcerative colitis. CD: Crohn’s disease.
